# Supplementary material for: Realizing high-power and high-capacity zinc/sodium metal anodes through interfacial chemistry regulation
Source: Nat Commun. 2021 May 25;12:3083. doi: 10.1038/s41467-021-23352-0 (PMC8149847; doi:10.1038/s41467-021-23352-0)
Supplement: Supplementary file 1 — Supplementary Information [file 41467_2021_23352_MOESM1_ESM.pdf]

Supplementary Information for

Realizing high-power and high-capacity zinc/sodium metal anodes through interfacial chemistry regulation

Zhen Hou[1], Yao Gao[1], Hong Tan[1], Biao Zhang*[1,2,3]

[1]*Department of Applied Physics, Research Institute for Smart Energy, The Hong Kong Polytechnic University, Hung Hom, Hong Kong, China.*

[2]*The Hong Kong Polytechnic University Shenzhen Research Institute, Shenzhen, China.*

[3]*Guangdong-Hong Kong-Macao Joint Laboratory for Photonic-Thermal-Electrical Energy Materials and Devices, Research Institute for Smart Energy, The Hong Kong Polytechnic University, Hung Hom, Hong Kong, China.*

Supplementary Note 1: Analysis of electric field distribution

The Ansys electrical conduction models were adopted to conduct the FEM simulation of the influence of the conductor layer modified separator on the electric field distribution. The FEM simulation was carried out in a rectangle area, the size of which is 8 μm by 5.8 μm. The pristine separator was modeled as a sieve plate with a thickness of 3.8 μm. The sieve plate is composed of rectangular channels with an aperture of 1.0 μm and a pore spacing of 1.0 μm. The conductor coating is modeled as a thin film with a thickness of 0.2 μm (Supplementary Fig. 1). The meshing grid adopts the four-node element whose size is controlled to 0.05 μm. The potential difference between cathode and anode was set as 0.1 V. The electrical conductivity of anode/cathode and separator was 5.81×10^{7} and 1.00×10^{-7} S m^{-1}, respectively. The electrical conductivity of Sn and electrolyte was 9.17×10^{5} and 1.00 S m^{-1}, respectively.

In both the pristine separator and the modified separator, we assumed an electrolyte layer between the separator and the anode, rather than allowing them to be in direct contact. This is because the surfaces of the anode and the separator are not absolutely smooth, which would result in them being in partial rather than full contact, even under external pressure. There are two areas between anode and modified separator, one is non-contact areas (situation 1 in Supplementary Fig. 2) and the other is contact areas (situation 2 in Supplementary Fig. 2). For situation 1, the uniform electric field distribution is achieved due to equipotential surface of conductive layer (as shown in Fig. **1**b). Therefore, Zn metal could be evenly deposited on anode during Zn deposition (as shown in the SEM images in Figs. **4**c, d). For situation 2, Zn metal would be preferentially deposited on modified separator due to its better zincophilicity than anode during Zn deposition (Fig. **1**d), which avoids the short circuit caused by Zn growth at anode tips.

The portion of the conductor coating that is in direct contact with the anode surface will have the same potential as the anode, since the conductor coating is not a complete and continuous whole (as shown in Supplementary Fig. 3 and the SEM images in Fig. **3**b). The non-contact areas of the conductor coating have different potentials. What we

simulate here is the electric field distribution in the non-contact region between the conductor coating and the anode, so the predefined boundary conditions are reasonable. It should be noted that even though the conductor coating is composed of multiple independent regions, it would be of great significance in improving the overall performance of the batteries if the electric field distribution of each local region can be homogenized.

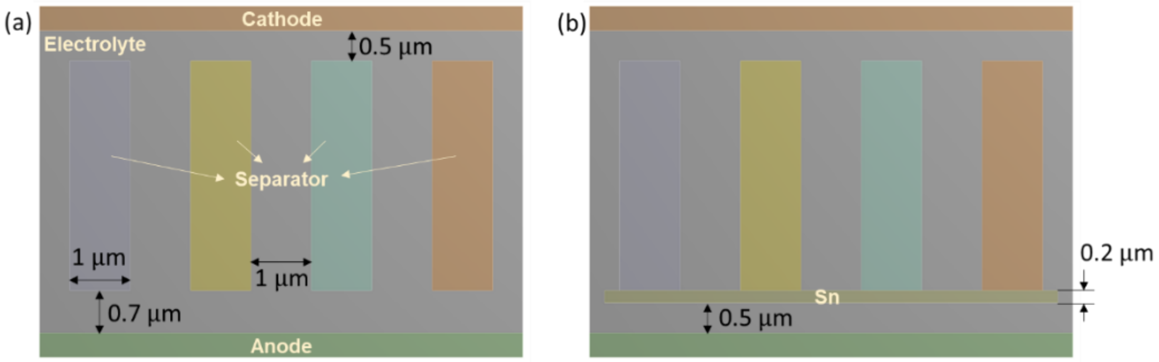

Supplementary Fig. 1 Finite element method (FEM) models for (**a**) pristine separator and (**b**) modified separator.

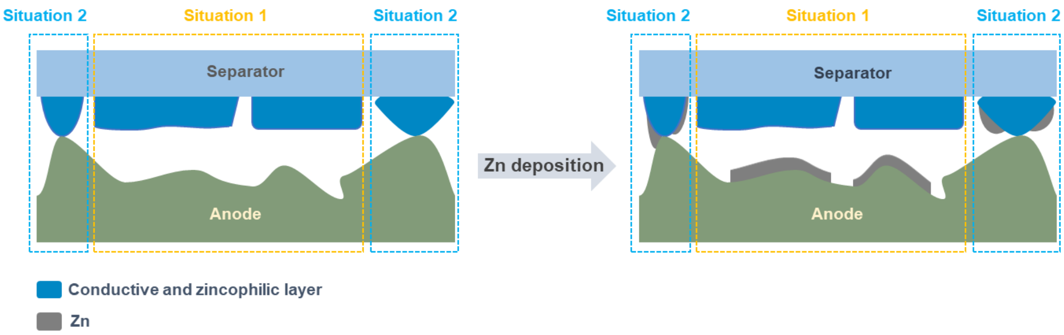

Supplementary Fig. 2 Two situations for modified separator during Zn deposition.

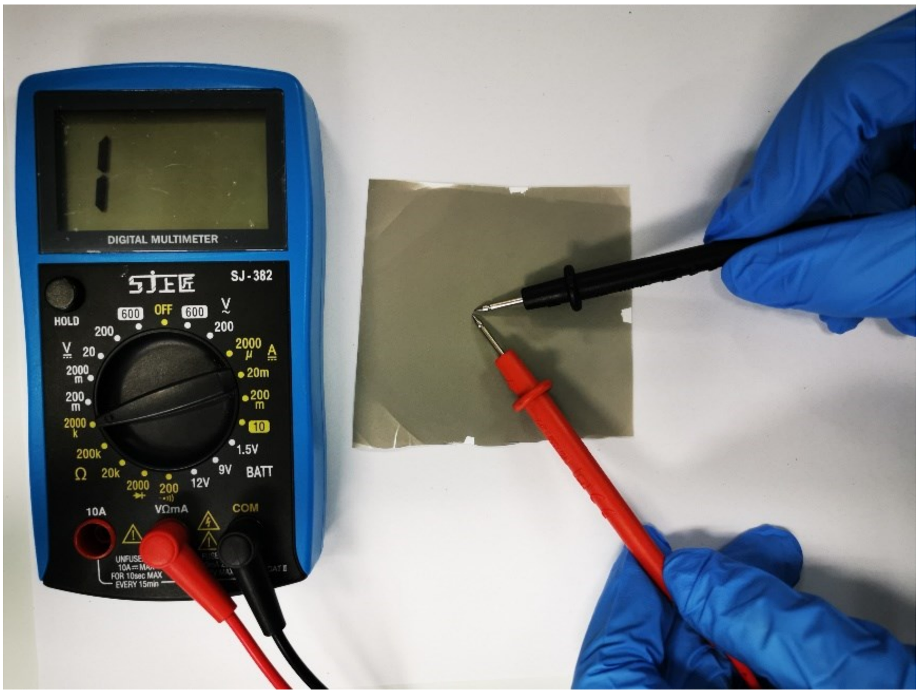

Supplementary Fig. 3 The resistance of modified separator after sputtering 1 min. The resistance of modified separator is too high to be detected because the conductor coating is not a complete and continuous whole.

Supplementary Note 2: Nucleation overpotential on various substrates

We investigate the nucleation overpotential (η) of Zn on different substrates (Supplementary Fig. 4). The η of SP-Ti current collector is 38 mV, which is comparable to the 44 mV of the bare Ti current collector. This indicates that the influence of Super P on the nucleation overpotential is minor. The plating potential for Zn^{2+} is around -0.02 V on Sn-Ti current collector. This value represents an overpotential required for overcoming the barrier to reduce Zn^{2+} into Zn metal, and the involved reaction is $Zn^{2+} + 2e^- \rightarrow Zn$.

CV is adopted to reveal the Zn-Sn alloy process (Supplementary Fig. 5). The rapidly increased oxidation current after ~0.6 V is attributed to the oxidation process of Sn metal, which agrees with its redox potential (0.62 V vs. Zn). As known, Ti is an electrochemically inert current collector for Zn batteries. Therefore, these redox peaks are ascribed to the alloying and dealloying reactions between Sn and Zn. The value of ~0.28 V is required to reduce Zn^{2+} and form an alloy with Sn[1,2], and the involved reaction is: $xZn^{2+} + Sn + 2xe^- \rightarrow SnZn_x$. The strong affinity of Sn to Zn greatly decreases the energy barrier required for the reduction of Zn^{2+} towards Zn.

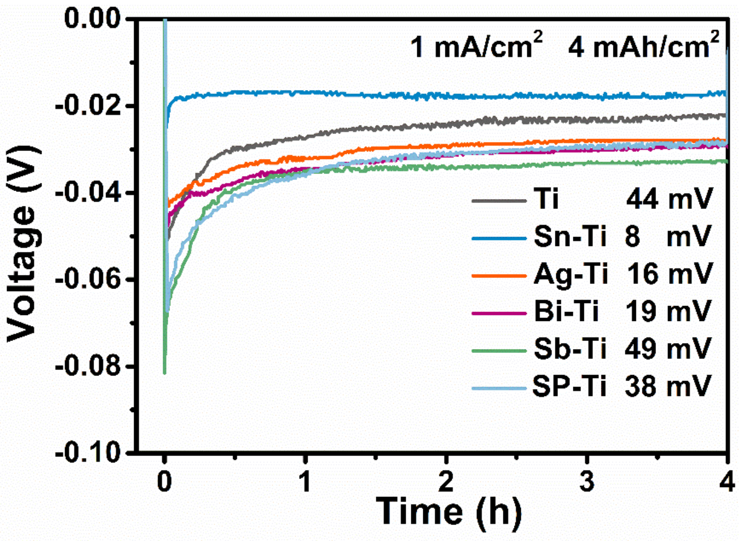

Supplementary Fig. 4 The nucleation overpotentials of Ti, metal-Ti and SP-Ti current collectors.

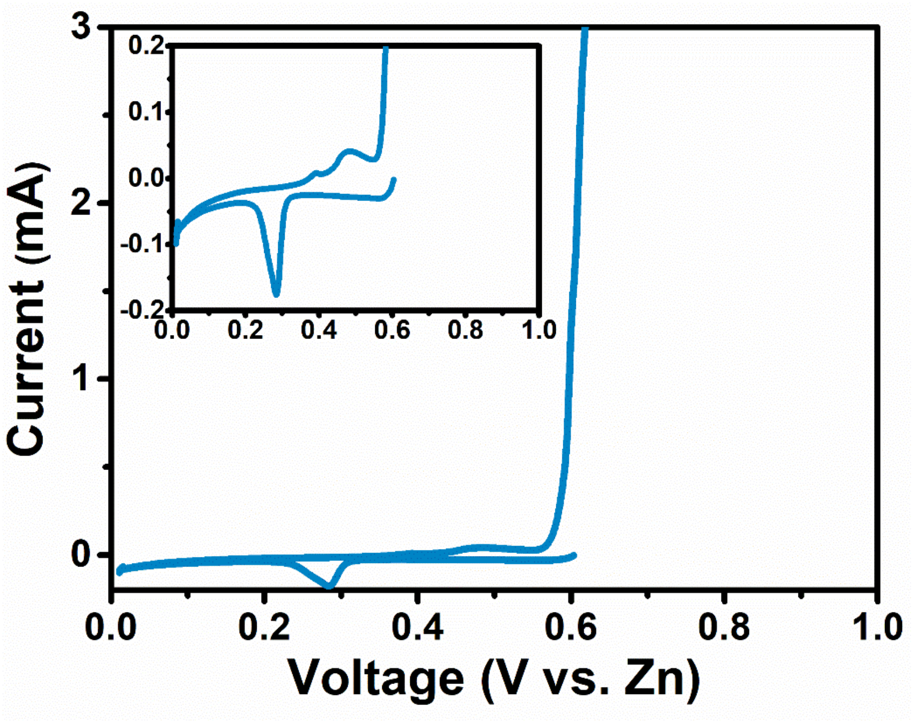

Supplementary Fig. 5 CV of Sn-Ti/Zn cell at a scan rate of 0.2 mV/s.

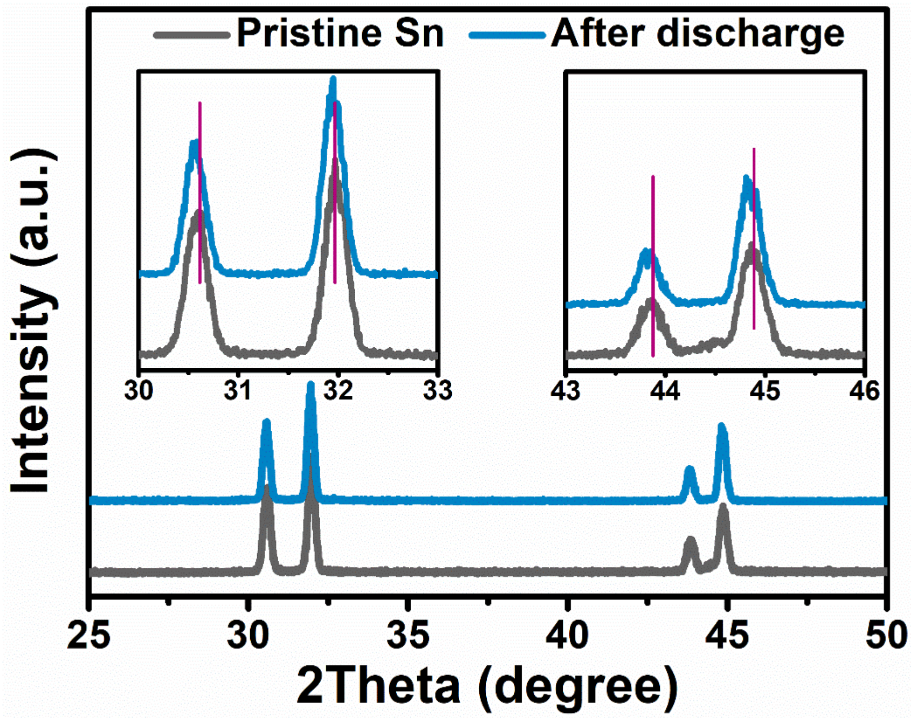

Supplementary Fig. 6. XRD spectra of for pristine Sn and after discharge.

Supplementary Note 3: Thickness control of Sn coating by magnetron sputtering

We examine the thickness controllability of magnetron sputtering by characterizing the thickness of films generated on the silicon wafer at different sputtering times using atomic force microscope (AFM). The sputtering time is set as 0.5, 1, 2 and 5 min, respectively. As shown in Supplementary Fig. 7, the thickness after 0.5 min sputtering time is ~20 nm, followed by ~40 nm (1 minute), ~80 nm (2 min) and ~170 nm (5 min). A linearly relationship between film thickness and sputtering time is found in the sputtering time range of 0-5 min, indicating that the film thickness can be precisely tuned using magnetron sputtering.

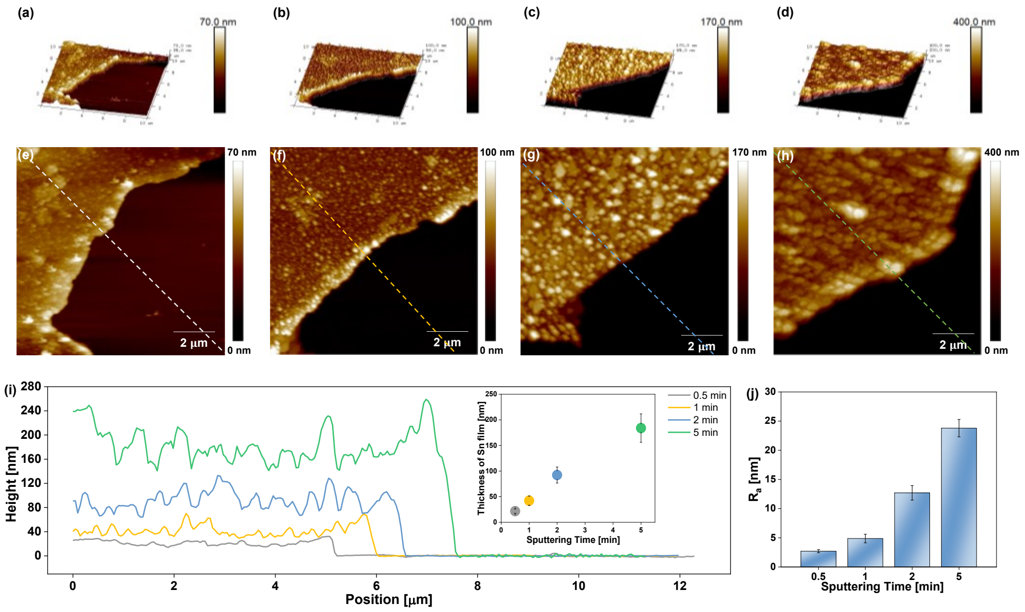

Supplementary Fig. 7 AFM images of Sn-coated separator with different sputtering time, (**a**, **e**) 0.5 minute, (**b**, **f**) 1 minute, (**c**, **g**) 2 minutes and (**d**, **h**) 5 minutes. (**i**) The thickness and (**j**) average roughness of Sn-coated separator with different sputtering time.

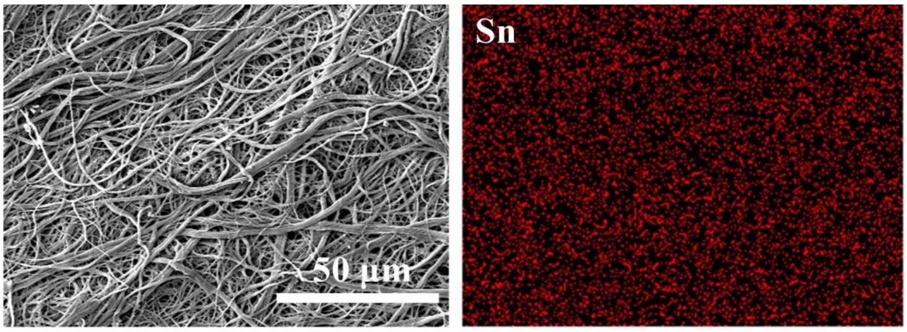

Supplementary Fig. 8 EDS mapping of Sn element on Sn-coated separator.

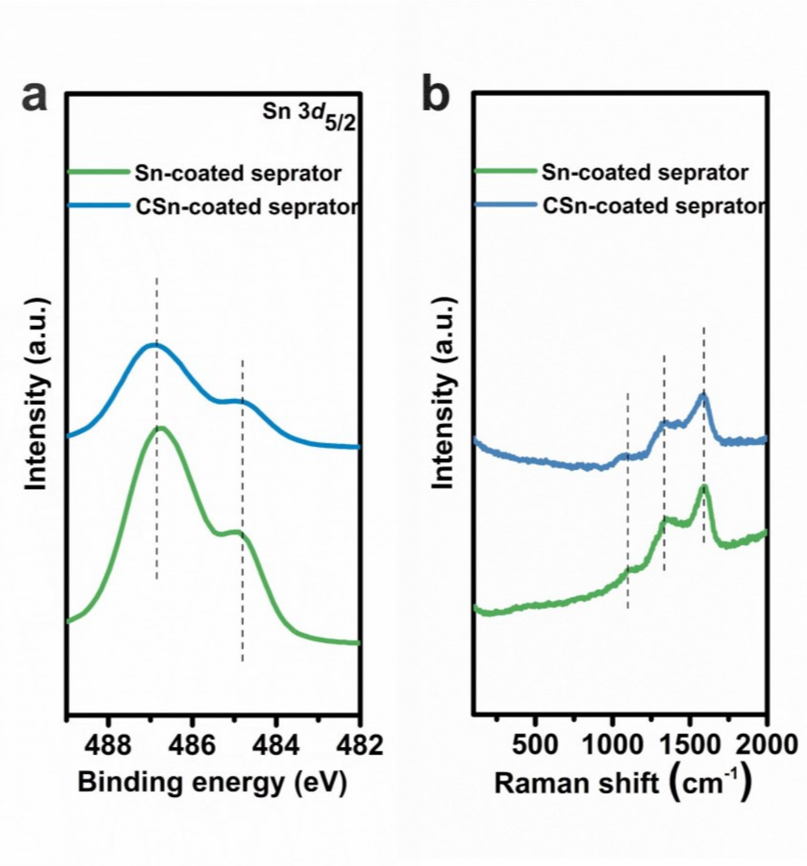

Supplementary Fig. 9 The (**a**) XPS and (**b**) Raman spectra of pristine Sn-coated separator and after 20-cycle Sn-coated separator (CSn-coated separator). The peak position of Sn $3d_{5/2}$ in XPS spectrum shows negligible shift after cycling in the electrolyte, confirming that the Sn layer on the separator is stable in the electrolyte. This is further proved by Raman spectra, where no new peaks are observed for the cycled Sn-coated separators compared to the pristine Sn-coated separator.

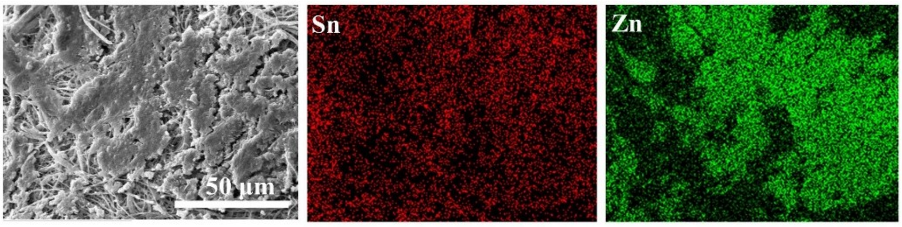

Supplementary Fig. 10 EDS mappings of Sn and Zn elements of Zn deposition on Sn-coated separator at 1 mA/cm^2 with a cycling capacity of 1 mAh/cm^2. The EDS mappings show that the Sn elements remain to be evenly distributed on the separator and the presence of Zn metal.

Supplementary Note 4: Reversibility of Zn anodes with different separators

The poor reversible Zn plating/stripping mainly originates from the formation of Zn dendrites with a large surface area, which promotes severe parasitic reactions between Zn metal anode and electrolyte. In addition, Zn dendrites may lose contact with the electrode and become "dead Zn" upon cycling. These would render low reversibility for Zn plating/stripping. As shown in Fig. **4**b, at a deposition capacity of 4 mAh/cm^2, loose structures with sharp tips are observed on Ti current collector using the pristine separator, which is not only detrimental to the reversibility of Zn metal anode but also might pierce the separator and cause a short circuit upon cycling. On the contrary, the Ti current collector in the cell using Sn-coated separator presents a greatly improved Zn deposition behavior. Specifically, dense and uniform Zn coatings are obtained (Fig. **4**d). The corresponding SEM images of Sn-coated separator also present a smooth structure of Zn metal (Fig. **4**f).

The dendrite-free morphologies can be attributed to two mechanisms as we mentioned previously. Firstly, for the region where the Sn coating is not in contact with the electrode, the equipotential property of Sn homogenizes the electric field distribution between the separator and the electrode (as evidenced by the FEM simulation in Figs. **1**a, b), allowing the Zn^{2+} flux to reach the electrode surface uniformly. Secondly, within the region where Sn coating is in contact with Ti current collector, Zn ions will prefer to deposit on Sn due to its zincophility instead of on Ti current collector, in turn realizing face-to-face growth of Zn. Both mechanisms will smooth the Zn deposition and the face-to-face grown Zn will merge at later stages to suppress the dendrite growth. Thus, an improved reversibility and stability of Zn plating/stripping could be achieved by using the Sn-coated separator (Supplementary Fig. 11).

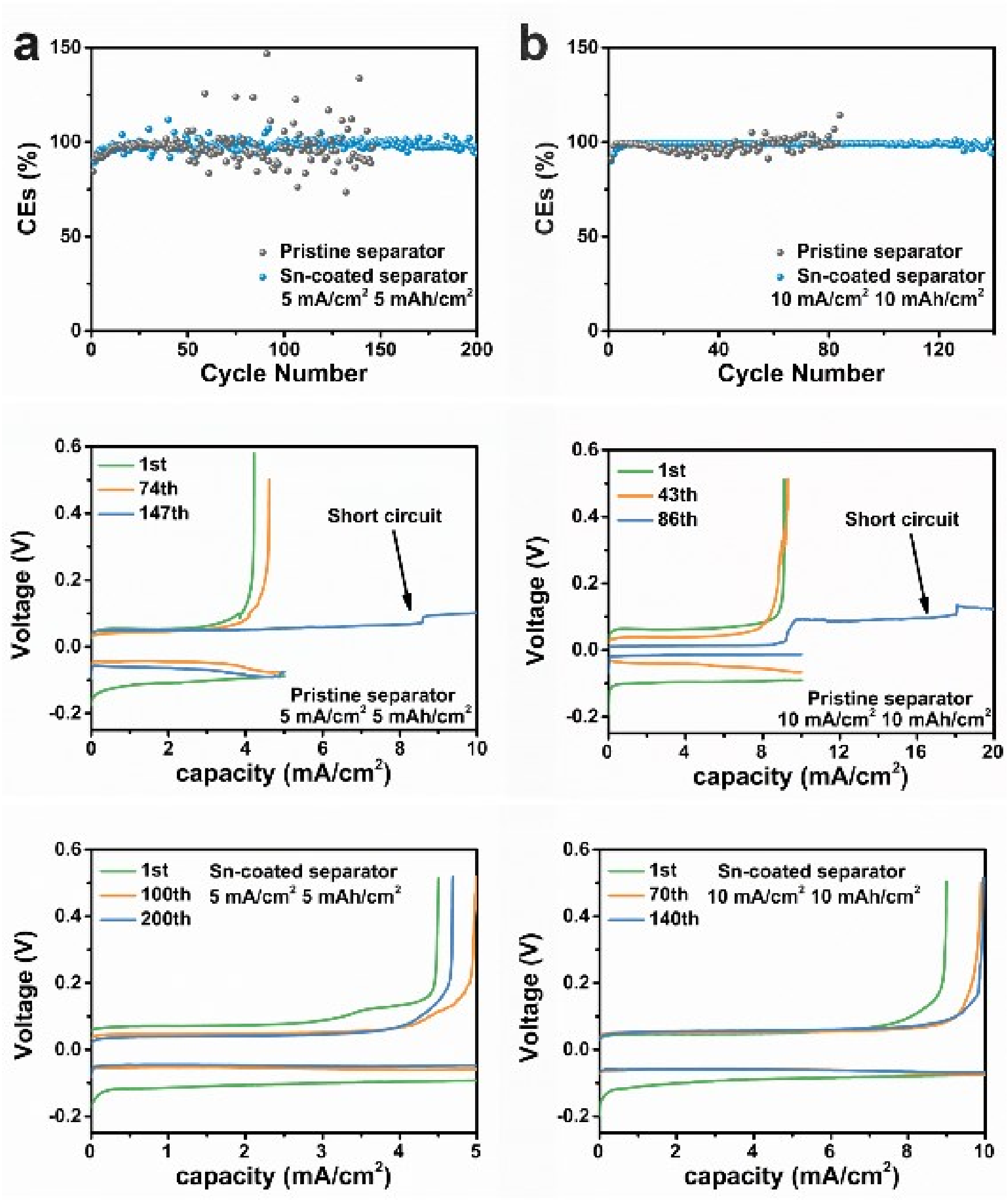

Supplementary Fig. 11 CEs of Ti/Zn cells and detailed deposition/stripping voltage curves using pristine separator and Sn-coated separator at (**a**) 5 mA/cm^2 and 5 mAh/cm^2 and (**b**) 10 mA/cm^2 and 10 mAh/cm^2.

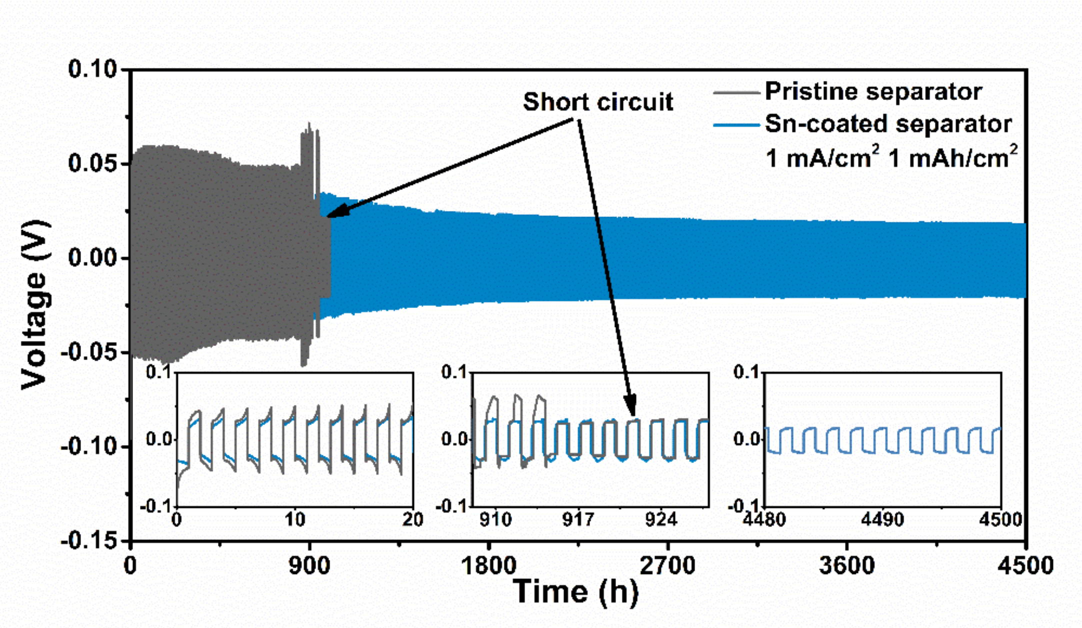

Supplementary Fig. 12 The cycling performance of Zn/Zn cells using pristine separator and Sn-coated separator tested at 1 mA/cm^2 and 1 mAh/cm^2. It is observed that the overpotential decreases with the cycles for Zn/Zn cells using both separators, which is attributed to the fact that the passivation layer on the pristine Zn foil is destroyed upon cycling[3].

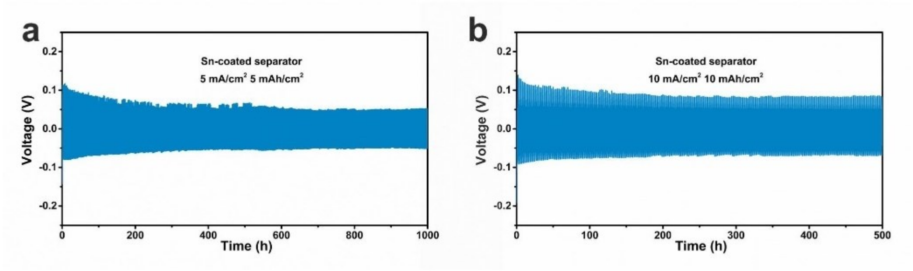

Supplementary Fig. 13 Reproducible experimental data for the cycling performances of Zn/Zn cells using pristine separator and Sn-coated separator tested at (**a**) 5 mA/cm^2 and 5 mAh/cm^2 and (**b**) 10 mA/cm^2 and 10 mAh/cm^2. The minor fluctuation in the voltage profiles is observed. We speculate the fluctuation is due to the concurrent deposition of Zn on both anode and Sn-coated separator.

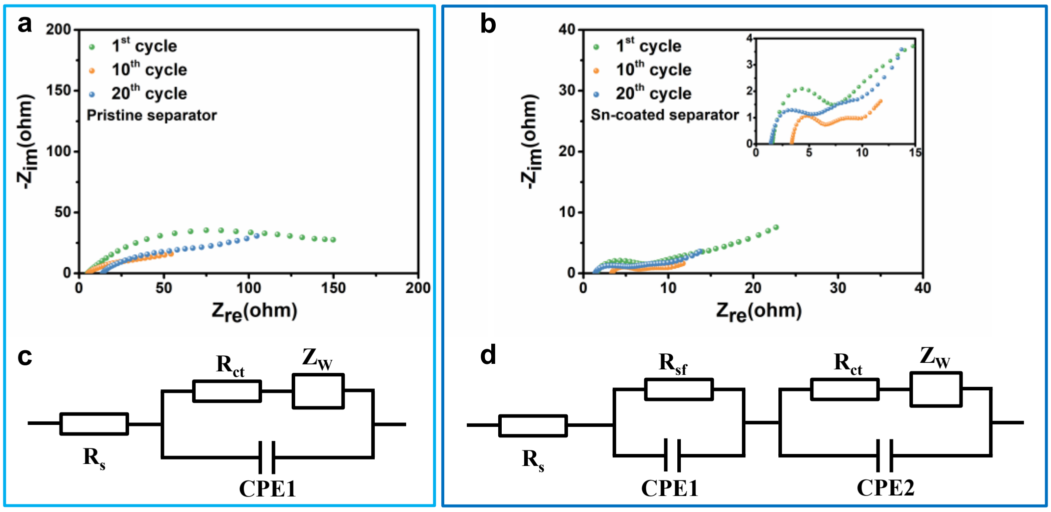

Supplementary Fig. 14 Nyquist plots using Zn/Zn cells with (**a**) pristine separator and (**b**) Sn-coated separator; Equivalent circuit models for EIS plots using Zn/Zn cells with (**c**) pristine separator and (**d**) Sn-coated separator.

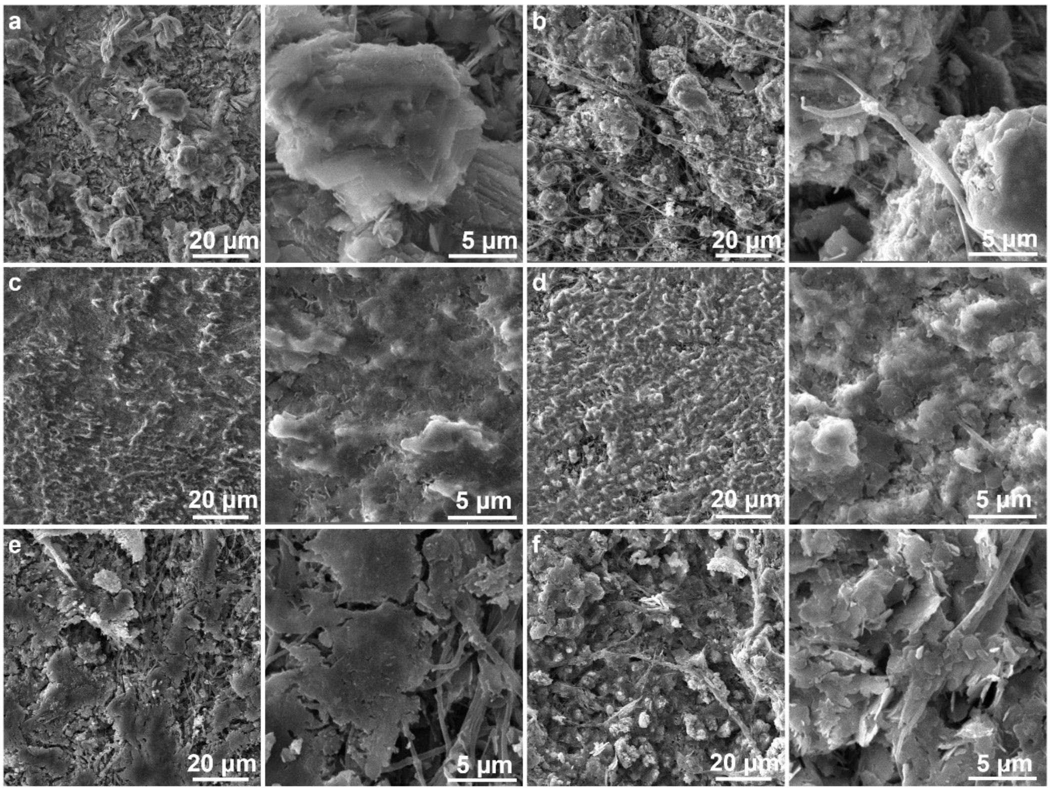

Supplementary Fig. 15 SEM images of the electrodes after Zn deposition at 5 mA/cm^2 and 5 mAh/cm^2. Cycled Zn using pristine separator after (**a**) 1 cycle and (**b**) 20 cycles; Zn using Sn-coated separator after (**c**) 1 cycle and (**d**) 20 cycles; Zn on cycled Sn-coated separator after (**e**) 1 cycle and (**f**) 20 cycles.

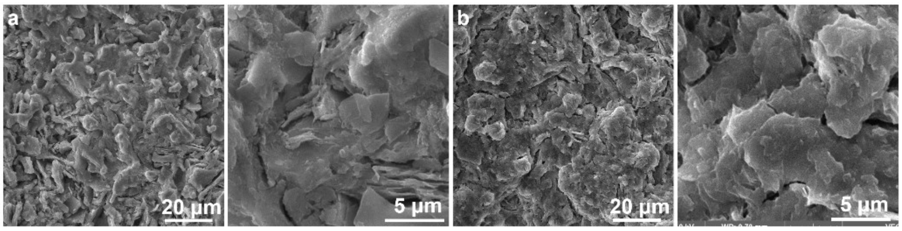

Supplementary Fig. 16 SEM images of (**a**) cycled Zn using Sn-coated separator and (**b**) cycled Zn on Sn-coated separator after 200 cycles at 5 mA/cm^2 and 5 mAh/cm^2. Zn deposition directions on both Zn anode and Sn-coated separator are parallel to the separator. Namely, the merging between Zn deposition from the anode and Sn-coated separator changes the Zn growth direction.

Supplementary Note 5: Comparison with different thickness of Sn coating and Ag-coating

To illustrate the working mechanism more clearly, we have done a series of comparative experiments. Sn-coated Zn foils (sputtering time: 1 min), Ag-coated separators (sputtering time: 1 min), Sn-coated separators (sputtering time: 0.5, 2, and 5 min) prepared using magnetron sputtering method.The electrochemical performance of Zn/Zn symmetric cells using these samples is evaluated.

As shown in Supplementary Fig. 17, cell with Sn-coated Zn foil shows a slightly improved cycle life compared with pristine Zn foil, but it is much shorter than that with Sn-coated separator. Although a zincophilic coating on the substrate is reported to regulate Zn nucleation and leads to relatively uniform Zn deposition. Such enhancement is generally limited to mild testing conditions. Supplementary Fig. 18 clearly displays the deposition/stripping process of Zn on Sn-coated Zn foil. Specifically, the zincophilic Sn coating on Zn foil facilitates more uniform Zn deposition during initial stages. However, under severe test conditions (high current and high cycling capacity), the uneven Zn^{2+} flux induced by the non-uniform electric field distribution greatly counteracts the effect of zincophilic coating, which triggers uneven Zn growth and leads to rapid short circuit after repeated cycles. For the case of Sn-coated separator, the Zn^{2+} flux reaches the electrode surface in an orderly manner driven by the uniform electric field, thus remaining effective at high currents and high cycling capacities.

In order to screen the ideal metal elements, we compare the zincophilicity of various metals using the parameter nucleation overpotential. Among Sn, Ag, Bi, and Sb, Ag is found to have the second highest Zn affinity after Sn (Supplementary Fig. 4). Therefore, Ag-coated separator is fabricated to verify the importance of zincophilicity for coating. As shown in Supplementary Fig. 19, the lifetime of the Zn/Zn cell with the Ag-coated separator is extended from 170 h to 460 h compared to the cell using pristine separator, while a stable cycle life of 1000 h is observed on cell using Sn-coated separator. This result demonstrates firstly that the zincophilic separator coating provides

improved cycling performance of the battery and secondly that the degree of improvement in cell performance is highly correlated with the degree of zincophilicity of the coating. Notably, a smaller overpotential is observed for the Ag-coated separator compared with the Sn-coated separator, probably because Ag has the highest electric conductivity among all the metals[4] (about an order of magnitude higher than Sn).

We assess the stability of Zn metal anodes using Sn-coated separators at different sputtering times. These samples are denoted as Sn-coated separator-X, where X represents the sputtering time (min). SEM images shows that bigger Sn particles and denser Sn layer are generated as the sputtering time increases (Supplementary Fig. 20). All Zn/Zn cells with these Sn-coated separators exhibit highly improved lifetime compared with the pristine separator at 5 mA/cm^2 and 5 mAh/cm^2, with the degree of improvement varying from one another (Supplementary Fig. 21). At the more rigorous test condition of 10 mA/cm^2 and 10 mAh/cm^2, the cycle life of Sn-coated separator-0.5 and Sn-coated separator-5 is much lower than that of Sn-coated separator-1 and Sn-coated separator-2 samples. For Sn-coated separator-0.5, the Sn layer is too thin to realize even electric field distribution and face-to-face growth of Zn, resulting in inferior electrochemical performance. Similar overpotentials are observed on these cells, indicating that the variation in thickness of nanoscale Sn layer has a negligible effect on the transport of Zn^{2+} through the modified separator. Namely, the difference of Zn^{2+} transport is not responsible for the shortened lifetimes of cells using Sn-coated separator-2 and Sn-coated separator-5. The 5 min sputtering time makes the Sn layer on the separator a continuous whole, which is indicated by the small resistance value between two points in the coating (Supplementary Fig. 23). This means that the Sn-coated separator-5 will have only one function of achieving face-to-face growth of Zn without the capability of homogenizing the electric field distribution, thus losing the competition when compared with Sn-coated separator-1 with two synergetic mechanisms. These results demonstrate that precise tuning of the Sn coating thickness at the nanoscale level to maximize the synergistic effect of these two mechanisms (equipotential surface and face-to-face Zn growth) is necessary to achieve significant

improvements in stability, especially under stringent test conditions.

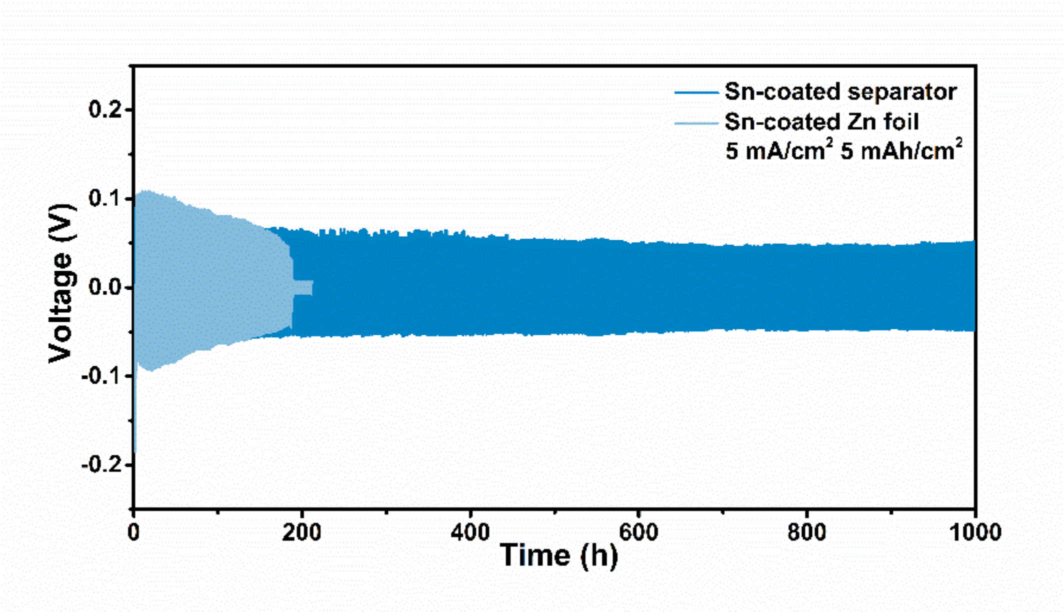

Supplementary Fig. 17 The cycling performances of cells using Sn-coated separator and Sn-coated Zn foil tested at 5 mA/cm^2 and 5 mAh/cm^2.

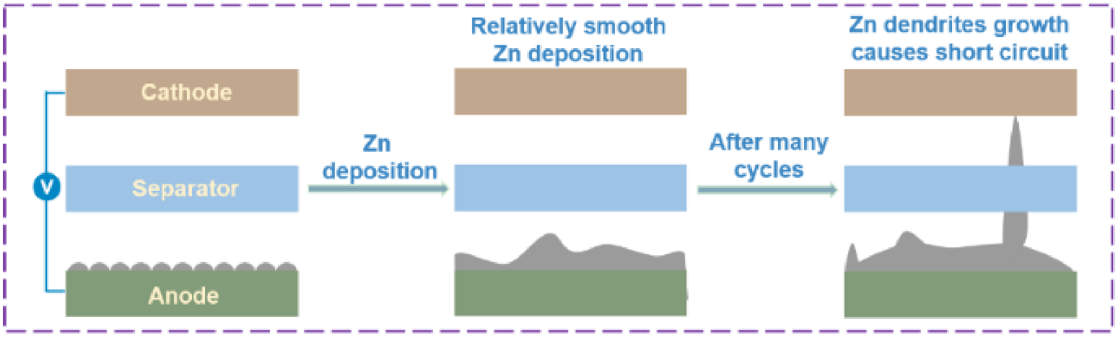

Supplementary Fig. 18 Schematic illustration of Zn deposition with Sn-coated Zn foil.

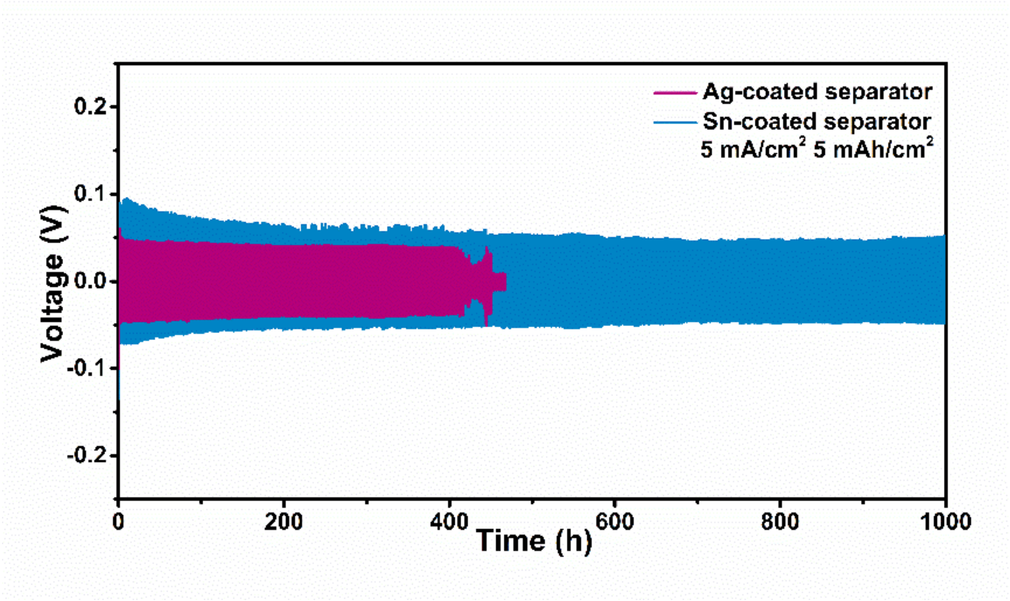

Supplementary Fig. 19 The cycling performances of cells using Sn-coated separator and Ag-coated separator tested at 5 mA/cm^2 and 5 mAh/cm^2.

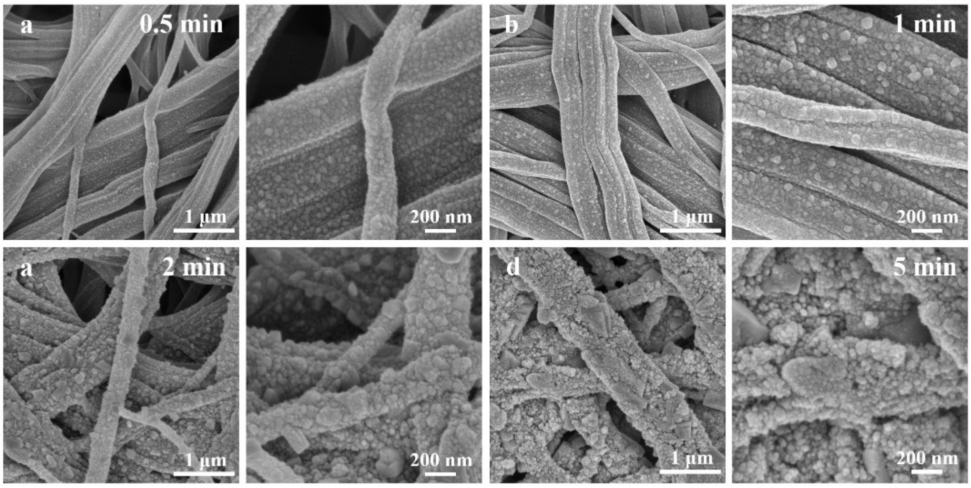

Supplementary Fig. 20 SEM images of (**a**) Sn-coated separator-0.5, (**b**) Sn-coated separator-1, (**c**) Sn-coated separator-2 and (**d**) Sn-coated separator-5.

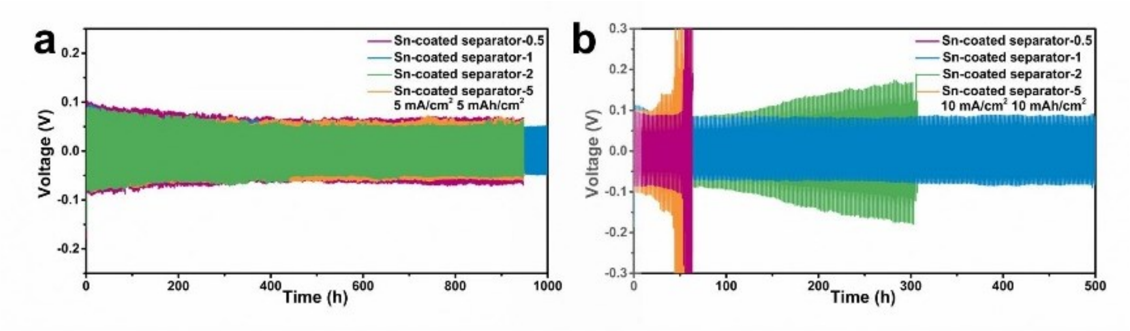

Supplementary Fig. 21 The cycling performances of cells using Sn-coated separator-X tested at 5 mA/cm^2 and 5 mAh/cm^2.

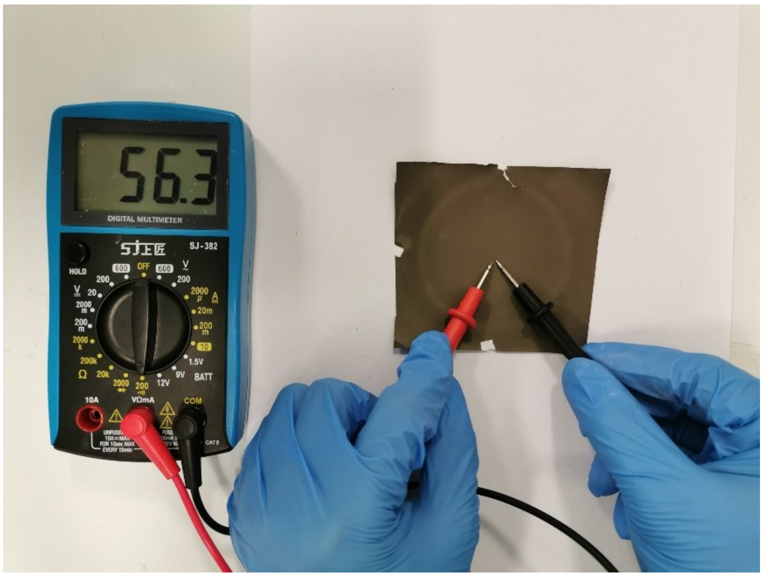

Supplementary Fig. 22 The resistance of Sn-coated separator-5.

Supplementary Note 6: Comparison with classic coating technique

Sn-coated separator and Zn foil are also prepared using the doctor blade method with the minimum thickness grade for the scraper (25 μm), respectively. For convenience, the samples are marked as DSn-coated separator and DSn-coated Zn foil. As shown in Supplementary Figs. 23a-d, aggregation and non-uniform distribution of Sn nanoparticles are observed on both the DSn-coated separator and DSn-coated Zn foil. Besides, the separator and Zn foil are completely covered by the Sn layer due to their micron thicknesses of ~12 μm (Supplementary Figs. 23e, f).

The cycling performance of Zn/Zn cells with Sn-coated and DSn-coated separators is compared at 5 mA/cm^2 and 5 mAh/cm^2. The cell using the DSn-coated separator shows an enormous increase in voltage potential after only ~40 h, which is even worse than that using the pristine separator. By contrast, stable cycle life of 1000 h is achieved for the cell using the Sn-coated separator under the same condition. The much inferior performance of DSn-coated separator than the pristine separator should stem from the uneven distributed Sn on the separator that causes nonuniform Zn deposition. In addition, although we have chosen the minimum thickness grade for the scraper, the Sn layer on the DSn-coated separator still has a thickness of around 12 μm. The thick Sn layer probably hinders the transport of Zn^{2+} and results in an elevated overpotential.

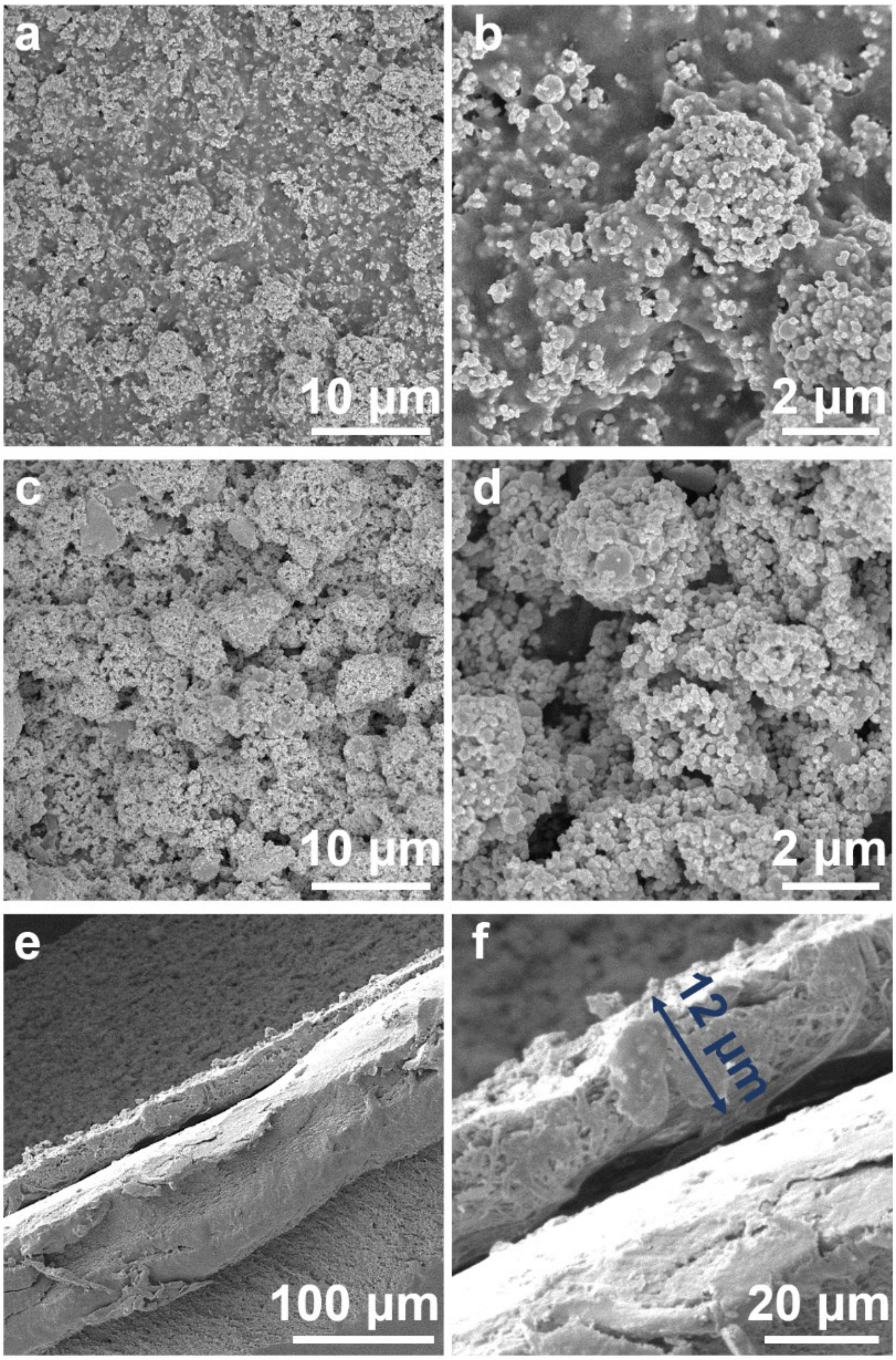

Supplementary Fig. 23 SEM images of (**a**, **b**) DSn-coated Zn foil and (**c**, **d**) DSn-coated separator. Cross section images of (**e**, **f**) DSn-coated Zn foil.

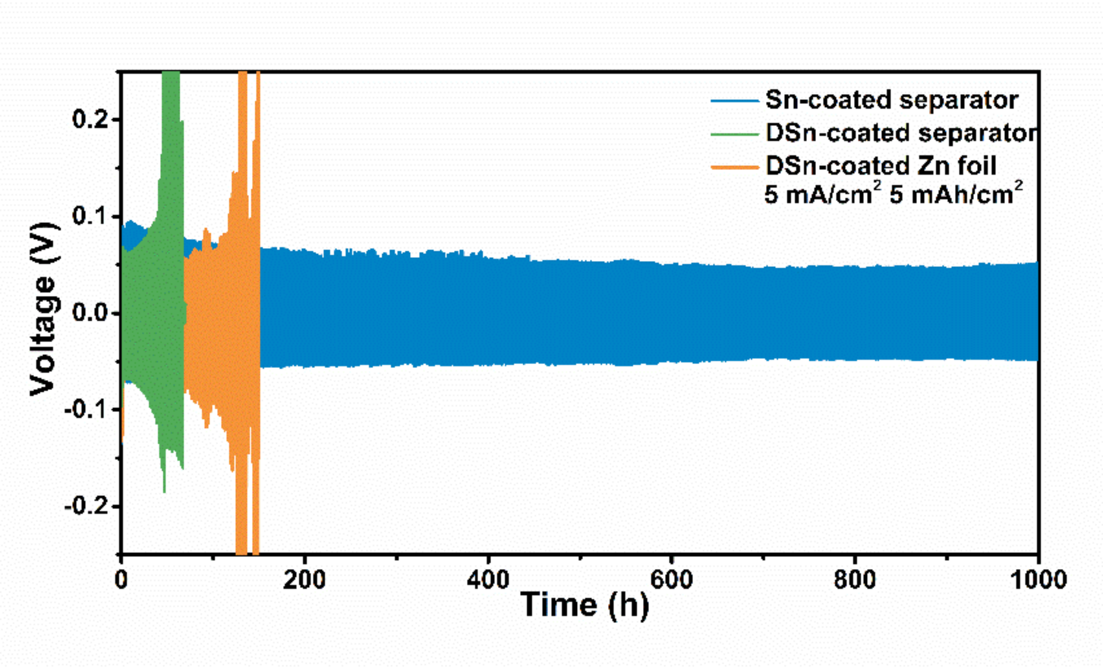

Supplementary Fig. 24 The cycling performances of cells using Sn-coated separator, DSn-coated separator and DSn-coated Zn foil tested at 5 mA/cm^2 and 5 mAh/cm^2.

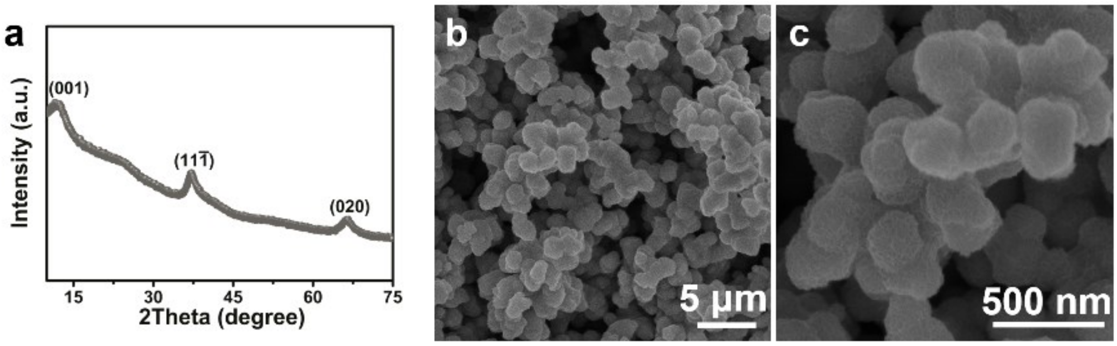

Supplementary Fig. 25 (**a**) The XRD spectrum and (**b**, **c**) SEM images of prepared MnO_2 materials. The diffraction peaks are consistent with the birnessite-type MnO_2 with a layered structure (JCPDS#43-1456)[5,6]. SEM images show that the morphology of such MnO_2 is flower-like nanospheres, which agrees with the literature[5].

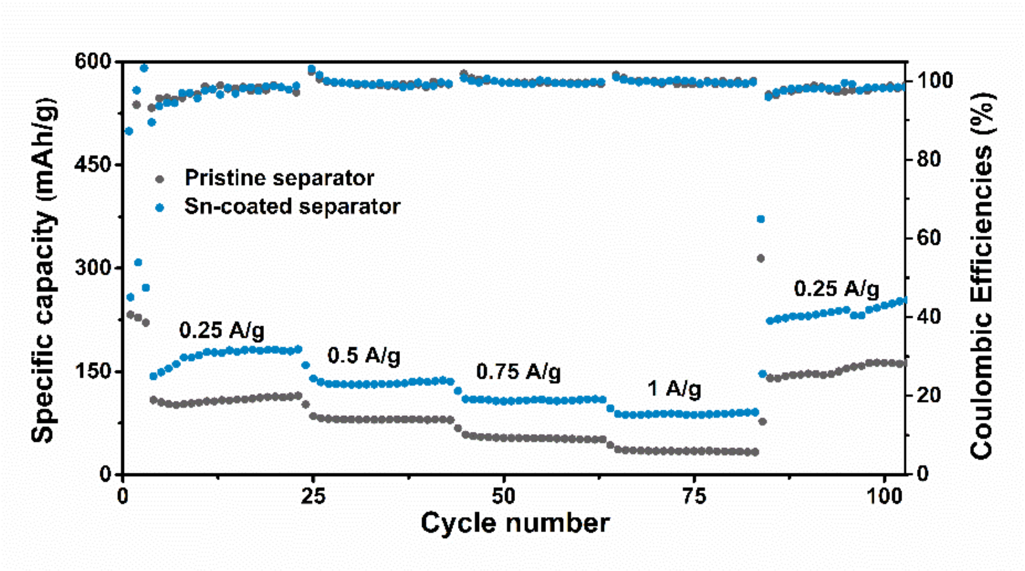

Supplementary Fig. 26 The rate performances of Zn||MnO_2 cells using pristine separator and Sn-coated separator.

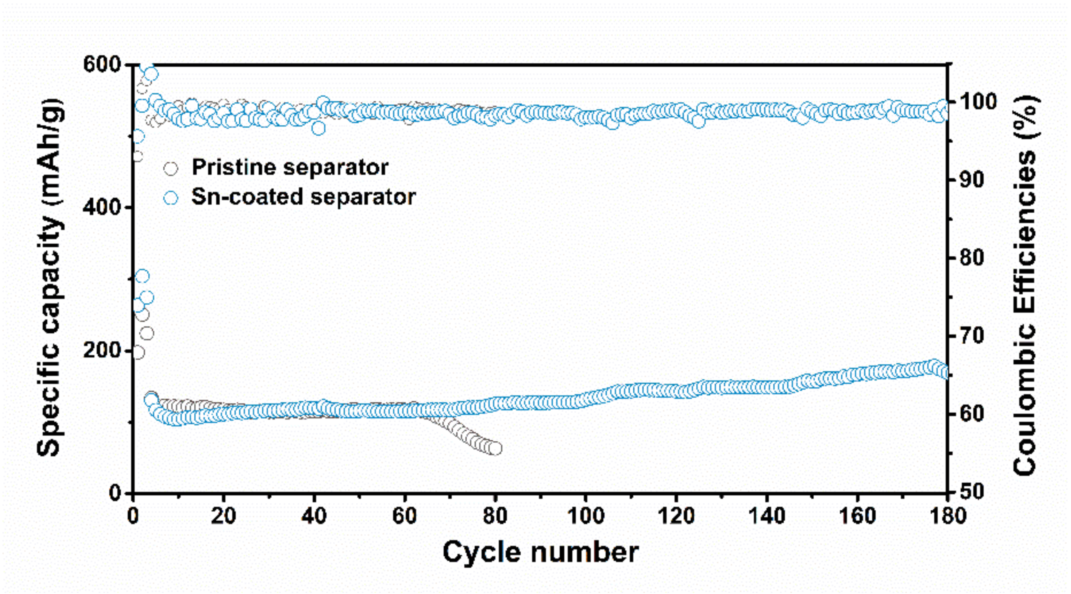

Supplementary Fig. 27 The cycling performances of Zn||MnO_2 cells using pristine separator and Sn-coated separator at 0.3 A/g with N:P ratios of 10:1.

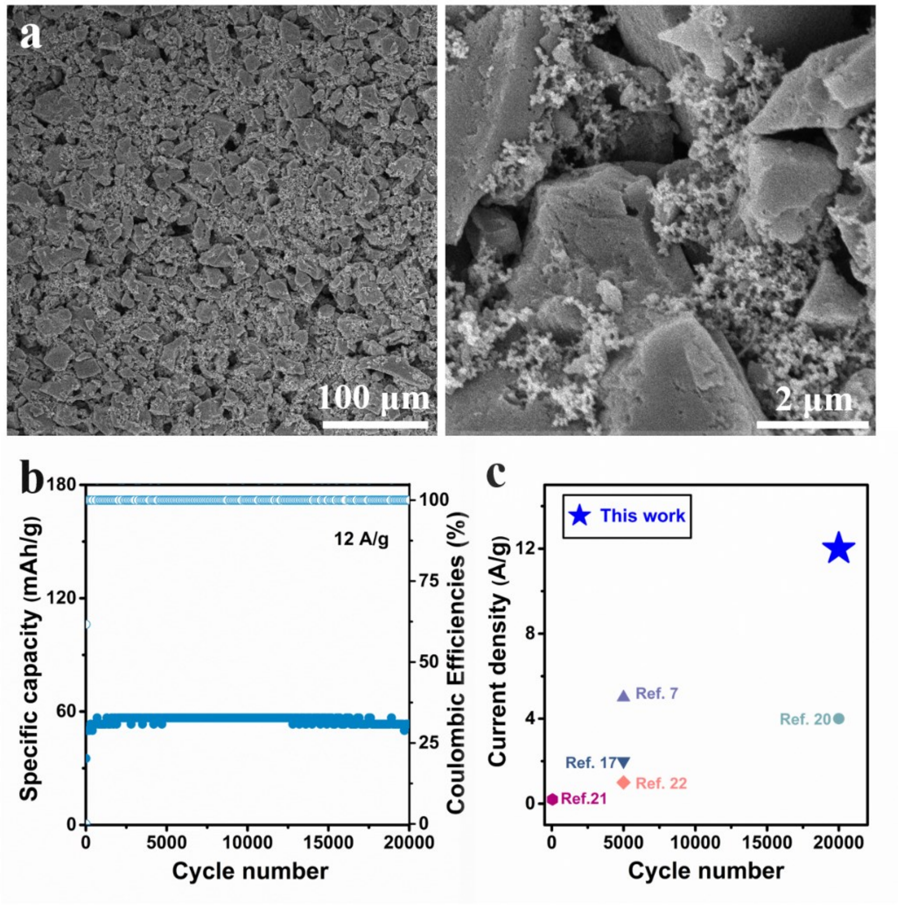

Supplementary Fig. 28 (**a**) SEM images of AC electrode. (**b**) Cycling performance of Zn/AC full cells using Sn-coated separator at 12 A/g. (**c**) Comparison of cycling performance (cycle number versus current density) in this work and previously reported works.

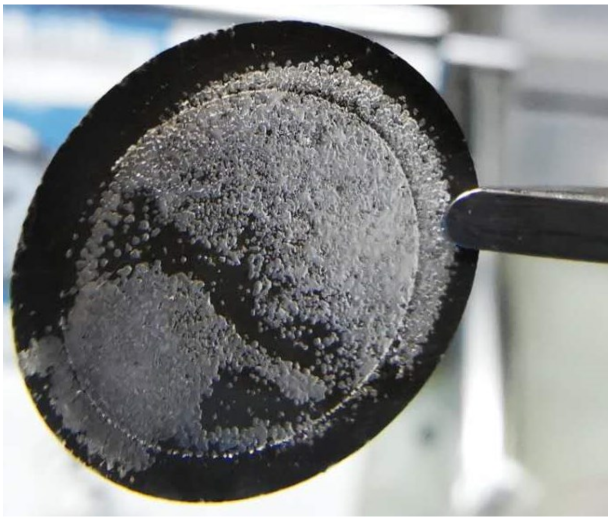

Supplementary Fig. 29 Optical photo of Na deposition on Sn-coated separator with 4 mAh/cm^2 at 1 mA/cm^2.

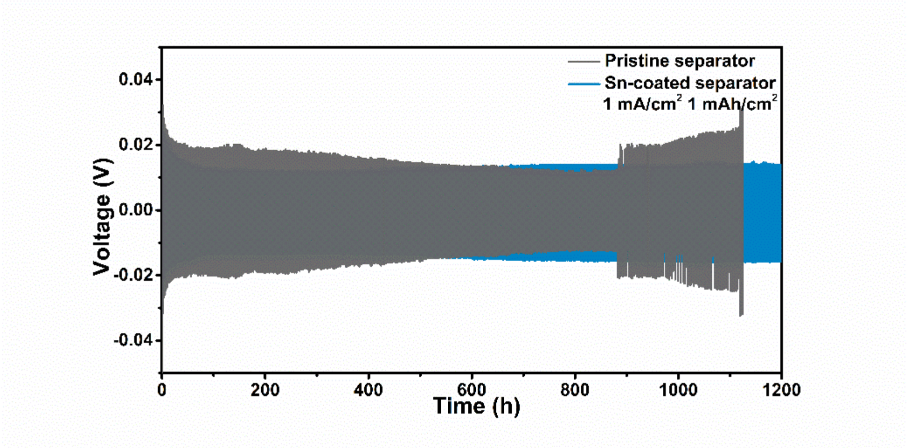

Supplementary Fig. 30 The cycling performances of Na/Na cells using pristine separator and Sn-coated separator tested at 1 mA/cm^2 and 1 mAh/cm^2.

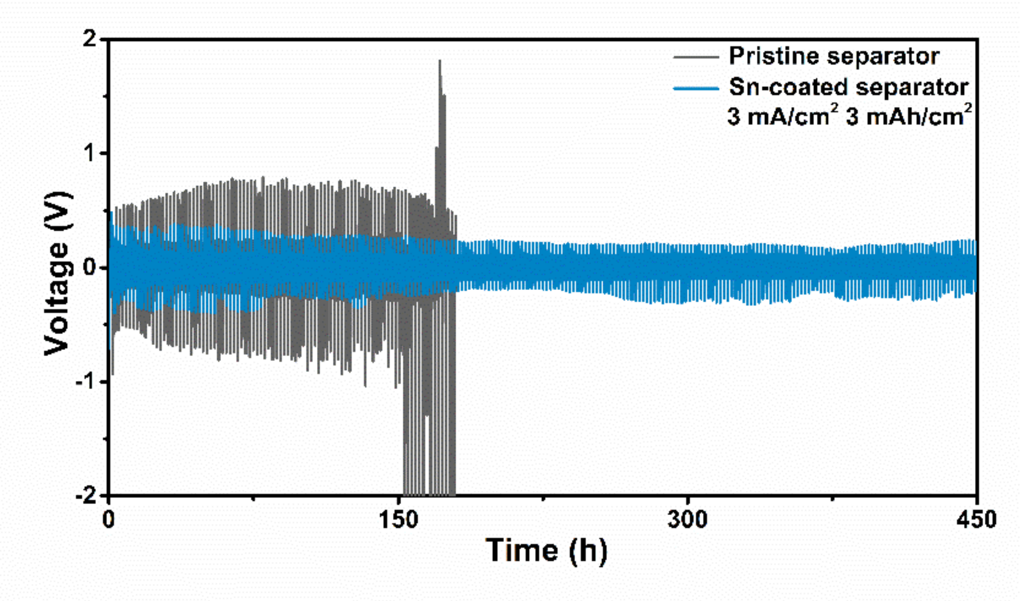

Supplementary Fig. 31 The cycling performances of K/K cells using pristine separator and Sn-coated separator are tested at 3 mA/cm^2 and 3 mAh/cm^2.

Supplementary Note 7: Effect of $MnSO_4$ electrolyte additive on the cyclic stability of Zn metal anodes

The cycling performance of Zn/Zn cells using 2 M $ZnSO_4$ + 0.2 M $MnSO_4$ as electrolyte is evaluated at 5 mA/cm^2 for 5 mAh/cm^2 and 10 mA/cm^2 for 10 mAh/cm^2. As shown in Supplementary Fig. 32, cells using Sn-coated separator with $MnSO_4$ additive could stably run for 900 h (5 mA/cm^2 and 5 mAh/cm^2) and 500 h (10 mA/cm^2 and 10 mAh/cm^2), which is similar with the case without $MnSO_4$ additive. Analogously, the stability using pristine separator with/without $MnSO_4$ additive are also comparable. These results demonstrate that the $MnSO_4$ additive has a negligible influence on Zn deposition/stripping behavior.

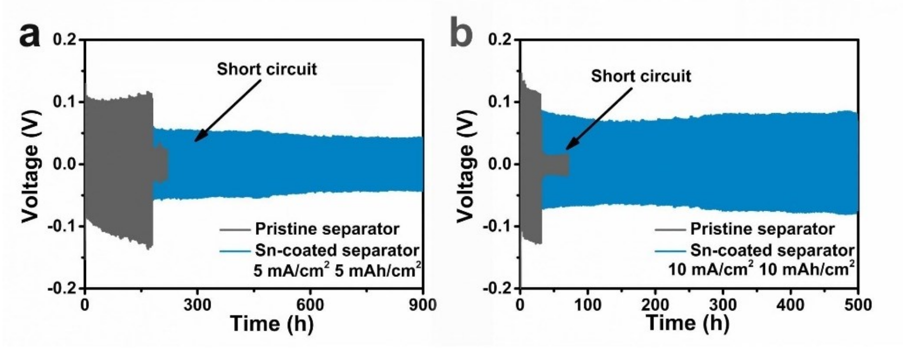

Supplementary Fig. 32 The cycling performance of cells using pristine separator and Sn-coated separator in 2 M $ZnSO_4$ + 0.2 M $MnSO_4$ electrolyte at (**a**) 5 mA/cm^2 and 5 mAh/cm^2 and (**b**) 10 mA/cm^2 and 10 mAh/cm^2.

Supplementary Table 1. Summary of electrochemical performances of Zn plating/stripping behaviors through modifying interfacial layer.

Interfacial layer	Current density (mA/cm^2)	Cycling capacity (mAh/cm^2)	Cycle life (h)	Cumulative capacity (mAh/cm^2)	Ref.
**Sn-coated separator**	**1**<br>**2**<br>**5**<br>**10**	**1**<br>**2**<br>**5**<br>**10**	**4500**<br>**3800**<br>**1000**<br>**500**	**4500**<br>**7600**<br>**5000**<br>**5000**	**This work**
Vertical graphene-coated separator	5<br>10	5<br>1	75<br>600	375<br>6000	7
Polyacrylonitrile separator	0.5	0.25	350	175	8
Reduced graphene oxide-coated Zn	10	2	200	2000	9
Carbon-coated Zn	10	1	100	1000	10
PVDF/TiO_2-coated Zn	8.85	8.85	250	2212.5	11
ZnO-coated Zn	5	1.25	500	2500	12
Polyamide-coated Zn	0.5<br>10	0.25<br>10	8000<br>80	4000<br>800	13
Kaolin-coated Zn	4.4	1.1	800	3520	14
Al_2O_3-coated Zn	1	1	500	500	15
TiO_2-coated Zn	1	1	150	150	16
Indium-coated Zn	1	1	500	500	17
MOF-coated Zn	0.5	0.5	700	350	18
$CaCO_3$-coated Zn	0.25	0.05	836	209	3
(0 0 1) facet-TiO_2-coated Zn	1	1	460	460	19

Supplementary Table 2. The fitting resistance results of Zn/Zn cells with pristine separator and Sn-coated separator after different cycles at 5 mA/cm^2 and 5 mAh/cm^2 by the equivalent circuits.

Samples	Resistance (Ω)	1st cycle	10th cycle	20th cycle
Pristine separator	R_s	4.97	5.18	13.56
	R_{ct}	132.20	42.14	73.69
Sn-coated separator	R_s	1.47	3.25	1.33
	R_{sf}	4.49	5.81	6.61
	R_{ct}	14.04	2.26	2.81

Supplementary Table 3. Summary of electrochemical performance of Zn metal batteries through modifying interfacial layer.

Modification	Cathode	Discharge capacity (mAh/cm^2)	Cycle Number	Capacity retention (%)	Ref.
**Sn-coated separator**	**MnO_2**	**~200**	**600**	**~100**	**This work**
Vertical graphene-coated separator	$V_2 0_5$	~150	1000	~75	[7]
Polyacrylonitrile separator	None				[8]
Reduced graphene oxide-coated Zn	$V_3O_7 \cdot H_2O$	~200	1000	~79	[9]
Carbon-coated Zn	NVP	~70	1000	~65	[10]
PVDF/TiO_2-coated Zn	MnO_2	~234	300	~100	[11]
ZnO-coated Zn	MnO_2	~212.9	500	~100	[12]
Polyamide-coated Zn	MnO_2	~176.1	1000	~88	[13]
Kaolin-coated Zn	MnO_2	~190	600	~86	[14]
Al_2O_3-coated Zn	MnO_2	~250	200	~74	[15]
TiO_2-coated Zn	MnO2	~150	1000	~85	[16]
$CaCO_3$-coated Zn	MnO_2	~185	1000	~86	[3]
(0 0 1) facet-TiO_2-coated Zn	MnO_2	~80	300	~84	[19]

Supplementary Table 4. Summary of electrochemical performance of Zn metal batteries using AC as cathode.

Modification	Current density (A/g)	Discharge capacity (mAh/cm^2)	Cycle Number	Capacity retention (%)	Ref.
**Sn-coated separator**	**12**	**~54**	**20000**	**~100**	**This work**
Vertical graphene-coated separator	5	~60	5000	~94	[7]
Indium-coated Zn	2	~70	5000	~86	[17]
MOF host	4	~58	20000	~72	[20]
Zincic perfluorinated sulfonic acid membrane	0.2	~138	50	None	[21]
Konjac glucomannan-coated Zn	1	~50	5000	~98.8	[22]

Supplementary References

1. Yan K*, et al.* Selective deposition and stable encapsulation of lithium through heterogeneous seeded growth. *Nat. Energy* **1**, 16010 (2016).

2. Tang S*, et al.* Stable Na plating and stripping electrochemistry promoted by in situ construction of an alloy-based sodiophilic interphase. *Adv. Mater*. **31**, e1807495 (2019).

3. Kang LT*, et al.* Nanoporous $CaCO_3$ coatings enabled uniform Zn stripping/plating for long-life zinc rechargeable aqueous batteries. *Adv. Energy Mater.* **8**, 1801090 (2018).

4. Liu Y, Ji C, Su X, Xu J, He X. Electromagnetic and microwave absorption properties of Ti_3SiC_2 powders decorated with Ag particles. *J. Alloys Compd.* **820**, 153154 (2020).

5. Wang J, Wang J-G, Liu H, Wei C, Kang F. Zinc ion stabilized MnO_2 nanospheres for high capacity and long lifespan aqueous zinc-ion batteries. *J. Mater. Chem. A* **7**, 13727-13735 (2019).

6. Han S-D*, et al.* Mechanism of Zn insertion into nanostructured δ-MnO_2: a nonaqueous rechargeable Zn metal battery. *Chem. Mater.* **29**, 4874-4884 (2017).

7. Li C, et al. Directly grown vertical graphene carpets as janus separators toward stabilized Zn metal anodes. *Adv. Mater.* **32**, e2003425 (2020).

8. Lee BS, et al. Dendrite suppression membranes for rechargeable zinc batteries. *ACS Appl. Mater. Interfaces* **10**, 38928-38935 (2018).

9. Shen C, et al. Graphene-boosted, high-performance aqueous Zn-ion battery.

ACS Appl. Mater. Interfaces **10**, 25446-25453 (2018).

10. Li W, Wang K, Zhou M, Zhan H, Cheng S, Jiang K. Advanced low-cost, high-voltage, long-life aqueous hybrid sodium/zinc batteries enabled by a dendrite-free zinc anode and concentrated electrolyte. *ACS Appl. Mater. Interfaces* **10**, 22059-22066 (2018).

11. Zhao R, et al. Redirected Zn electrodeposition by an anti-corrosion elastic constraint for highly reversible Zn anodes. *Adv. Funct. Mater.* **31**, 2001867 (2020).

12. Xie X, et al. Manipulating the ion-transfer kinetics and interface stability for high-performance zinc metal anodes. *Energy Environ. Sci.* **13**, 503-510 (2020).

13. Zhao Z, et al. Long-life and deeply rechargeable aqueous Zn anodes enabled by a multifunctional brightener-inspired interphase. *Energy Environ. Sci.* **12**, 1938-1949 (2019).

14. Deng C, et al. A sieve-functional and uniform-porous kaolin layer toward stable zinc metal anode. *Adv. Funct. Mate*r. **30**, 2000599 (2020).

15. He H, Tong H, Song X, Song X, Liu J. Highly stable Zn metal anodes enabled by atomic layer deposited Al_2O_3 coating for aqueous zinc-ion batteries. *J. Mater. Chem. A* **8**, 7836-7846 (2020).

16. Zhao K, et al. Ultrathin surface coating enables stabilized zinc metal anode. A*dv. Mater. Interfaces* **5**, 1800848 (2018).

17. Han D, et al. A corrosion-resistant and dendrite-free zinc metal anode in aqueous systems. *Small* **16**, e2001736 (2020).

18. Cao L, Li D, Deng T, Li Q, Wang C. Hydrophobic organic electrolyte protected Zn anodes for aqueous Zn batteries. *Angew. Chem. Int. Ed.* **59**, 19292-19296 (2020).

19. Zhang Q*, et al.* Revealing the role of crystal orientation of protective layers for stable zinc anode. *Nat. Commun.* **11**, 3961 (2020).

20. Wang Z*, et al.* A metal-organic framework host for highly reversible dendrite-free zinc metal anodes. *Joule* **3**, 1289-1300 (2019).

21. Cui Y*, et al.* Quasi-solid single Zn-ion conductor with high conductivity enabling dendrite-free Zn metal anode. *Energy Stor. Mater.* **27**, 1-8 (2020).

22. Zou K*, et al.* Highly stable zinc metal anode enabled by oxygen functional groups for advanced Zn-ion supercapacitors. *Chem. Commun.* **57**, 528-531 (2021).
