# Supplementary material for: Realizing high-power and high-capacity zinc/sodium metal anodes through interfacial chemistry regulation
Source: Nat Commun. 2021 May 25;12:3083. doi: 10.1038/s41467-021-23352-0 (PMC8149847; doi:10.1038/s41467-021-23352-0)
Supplement: Supplementary file 3 — Description of Additional Supplementary Files [file 41467_2021_23352_MOESM3_ESM.docx]

**Description of Additional Supplementary Files**

File Name: Supplementary Movie 1

Description: Simulation of the electric field distribution in the cell with the pristine separator

File Name: Supplementary Movie 2

Description: Simulation of electric field distribution in the non-contact region between the modified separator and the anode
